# Editorial: Women in endocrinology 2021

**DOI:** 10.3389/fendo.2022.987727

**Published:** 2022-07-28

**Authors:** Claire M. Perks, Sally Radovick, Katherine Samaras

**Affiliations:** ^1^ Bristol Medical School, Department of Translational Health Sciences, University of Bristol, Bristol, United Kingdom; ^2^ Department of Pediatrics, Rutgers-Robert Wood Johnson Medical School, New Brunswick, NJ, United States; ^3^ Department of Endocrinology, St Vincent’s Hospital, Darlinghurst, NSW, Australia; ^4^ Clinical Obesity, Nutrition and Adipose Biology Lab, Clinical Science Pillar, Garvan Institute of Medical Research, Darlinghurst, NSW, Australia; ^5^ St Vincent’s Clinical School, Faculty of Medicine, University of New South Wales, Darlinghurst, NSW, Australia

**Keywords:** women, endocrinology, obesity, reproductive years, international day of women

‘The International Day of Women and Girls in Science,’ was celebrated on the 11^th^ of February this year, ‘to inspire, promote and encourage equal access to science, gender equality and empowerment’ (1). This research Topic was launched to provide such a forum in which to promote and celebrate our women researchers in Endocrinology internationally. Despite such efforts to recognise and acknowledge the essential and pioneering work of women in science, there is still work to be done. This was highlighted in a Brief Research Report written by Raven and McCormack entitled ‘Female Representation: Australian Diabetes and Endocrinology Societies’ that performed an audit of Australian diabetes and endocrinology societies between 2016 and 2020, and their respective annual scientific meetings, in relation to gender participation. Whilst there was equal representation of males and females amongst speakers and session chairs, less women were assigned to the more prestigious roles of plenary speakers and society council members.

The prevalence of obesity is increasing globally, and the ensuing endocrine alterations are having a huge impact on health. Notably, the COVID-19 pandemic led to worsened levels of obesity and was cited as one of the risk factors associated with severe disease and death. Tchang et al. authored an interesting paper on how telemedicine, implemented during the pandemic, influenced weight loss outcomes and management. Her retrospective observational study suggested that telemedicine was similarly effective to in person visits for weight loss outcomes and therefore for specific groups this may be a good strategy going forward.

The impact of obesity was highlighted in three Original Research Papers authored by Neal et al., Tseng et al. and Li et al.. Neal et al. examined the effect of obesity class on maternal and perinatal outcomes and revealed that obesity class was independently associated with the incidence of large for gestational age (LGA) in the neonate, irrespective of maternal factors. Tseng et al. determined that obesity affected sleep and psychiatric disorders associated with irritable bowel syndrome (IBS) in women with polycystic ovary syndrome (PCOS) and suggested that screening and management of IBS and obesity for those with PCOS was warranted. Additionally, Li et al. reported that pregnant women who were obese and exhibited gestational abnormal glucose metabolism were at far higher risk of poor pregnancy outcomes than with women with either condition in isolation.In contrast to the impact of obesity in relation to LGA, Wang et al. found that in underweight women, whose total cholesterol levels were above the Institute of Medicines’ recommendations, there was a higher risk for LGA, compared with women of a normal weight or who were overweight. Taken together, these papers argue for heightened efforts in obesity prevention and treatment through a woman’s reproductive years. The findings of these papers indicated that there is no justification for inaction or neglect of a women’s obesity simply because cardiometabolic complications are yet to develop, our current therapeutic model.

The impact of stress on the hypothalamic-pituitary-adrenal (HPA) axis is key to striking a balance between homeostasis and can impact many downstream physiological processes including metabolic diseases such as obesity, mood disorders and fertility. This important relationship was explored further by two papers in this Research Topic. Moraes et al. and her team compared the effects of dissimilar stressors in rats (cold, movement restraint and predator odour) of different magnitudes on the activation of the hypothalamic-pituitary-adrenal (HPA) axis compared with the effect of paradoxical sleep deprivation (PSD). This study revealed that the HPA response to PSD was most similar to that induced by predator odour but was distinctive as it was the only stressor to increase the activity of the vasopressin system. Lin et al. pursued an optogenetic approach in mice to selectively stimulate kisspeptin production from the arcuate nucleus (ARC) to examine the effect on the surge of luteinizing hormone (LH) in response to different hormonal stimuli. The group determined that ARC kisspeptin did play a role in amplifying the LH surge, but this was dependent on levels of oestrogen and progesterone.

In conclusion, the “Women in Endocrinology 2021” Research Topic has provided a platform for enabling important contributions from female investigators in the field to be highlighted. In particular, data highlighted the ongoing disparities within the field of the endocrinology workforce and opportunities to improve the outcome for women during their reproductive years through attention to obesity. We look forward to future opportunities and forums in which to celebrate, promote and inspire women in the medical sciences and for measures to ensure parity is achieved.

## Author contributions

All authors listed have made a substantial, direct, and intellectual contribution to the work and approved it for publication.

## Conflict of interest

The authors declare that the research was conducted in the absence of any commercial or financial relationships that could be construed as a potential conflict of interest.

## Publisher’s note

All claims expressed in this article are solely those of the authors and do not necessarily represent those of their affiliated organizations, or those of the publisher, the editors and the reviewers. Any product that may be evaluated in this article, or claim that may be made by its manufacturer, is not guaranteed or endorsed by the publisher.

## References

[1] United Nations International day of women and girls in science. Available at: https://www.un.org/en/observances/women-and-girls-in-science-day (Accessed 5/6/22).

